# Injection Molding and Near-Complete Densification of Monolithic and Al_2_O_3_ Fiber-Reinforced Ti_2_AlC MAX Phase Composites

**DOI:** 10.3390/ma14133632

**Published:** 2021-06-29

**Authors:** Sylvain Badie, Rimy Gabriel, Doris Sebold, Robert Vaßen, Olivier Guillon, Jesus Gonzalez-Julian

**Affiliations:** 1 Institute of Energy and Climate Research, Materials Synthesis and Processing (IEK-1), Forschungszentrum Jülich GmbH, 52425 Jülich, Germany; r.gabriel@fz-juelich.de (R.G.); d.sebold@fz-juelich.de (D.S.); r.vassen@fz-juelich.de (R.V.); o.guillon@fz-juelich.de (O.G.); j.gonzalez@fz-juelich.de (J.G.-J.); 2 Department of Ceramics and Refractory Materials, Institute of Mineral Engineering, RWTH Aachen University, 52064 Aachen, Germany; 3 Jülich Aachen Research Alliance, JARA-Energy, 52425 Jülich, Germany

**Keywords:** Ti_2_AlC, MAX phases, powder injection molding, ceramic matrix composites, powder bed sintering, spark plasma sintering

## Abstract

Near-net shape components composed of monolithic Ti_2_AlC and composites thereof, containing up to 20 vol.% Al_2_O_3_ fibers, were fabricated by powder injection molding. Fibers were homogeneously dispersed and preferentially oriented, due to flow constriction and shear-induced velocity gradients. After a two-stage debinding procedure, the injection-molded parts were sintered by pressureless sintering at 1250 °C and 1400 °C under argon, leading to relative densities of up to 70% and 92%, respectively. In order to achieve near-complete densification, field assisted sintering technology/spark plasma sintering in a graphite powder bed was used, yielding final relative densities of up to 98.6% and 97.2% for monolithic and composite parts, respectively. While the monolithic parts shrank isotropically, composite assemblies underwent anisotropic densification due to constrained sintering, on account of the ceramic fibers and their specific orientation. No significant increase, either in hardness or in toughness, upon the incorporation of Al_2_O_3_ fibers was observed. The 20 vol.% Al_2_O_3_ fiber-reinforced specimen accommodated deformation by producing neat and well-defined pyramidal indents at every load up to a 30 kgf (~294 N).

## 1. Introduction

MAX phases (M = early transition metal, A = A-group element, X = C and/or N) have emerged as promising candidates for high-temperature applications [1], combining the characteristics from both metallic and ceramic materials [2], such as good thermal and electrical conductivities, damage tolerance, low density, thermal shock [3] and oxidation resistance [4]. Among Al-containing MAX phases, Ti_2_AlC stands out for its excellent long-term high-temperature oxidation resistance up to temperatures of around 1300 °C [5,6] and its chemical and thermal compatibility with thermal barrier coatings, such as YSZ [7].

Alongside ab initio studies to predict the existence of new MAX phases, extensive research has been dedicated, in the past three decades, to exploring new production/processing routes, investigating in-depth thermo-mechanical properties, and fine tuning synthesis parameters. However, the report of complex shape production via existing methods and the use of additive manufacturing is still in its early stages and has been barely explored for MAX phases. This partly explains why their transfer to industrial applications is—apart from Cr_2_AlC pantographs, which are used for high-speed trains in China [1]—still greatly limited. Even though cellular and lattice architectures [8,9,10,11], as well as bulk-injection molded components [12,13], have been successfully produced so far, there is still a lack of knowledge in this field.

Fast and reliable shaping techniques are required in the industry for mass production and cost reduction. Here, injection molding turned out to be a suitable method, allying these criteria with versatility in mold design and material combination. In particular, powder injection molding (PIM) has remained a powerful manufacturing technique to rapidly produce near-net-shape metallic or ceramic high-performance precision parts [14]. Not only was the injection molding of millimeter-sized monolithic Ti_2_AlC building blocks [13] investigated, but a study into the fabrication of Ti_3_SiC_2_, Ti_2_AlC and Ti_2_AlC/SiC_(f)_ components with high surface quality confirmed successful production by PIM [12]. In addition, this rapid manufacturing technique allows for producing composite parts with high homogeneity, aiming for higher temperature strength retention and mechanical properties than their monolithic counterparts.

In fact, several studies [15,16,17,18] into fiber and whisker-reinforced MAX phases demonstrated their superior thermomechanical response. Most often, the use of silicon carbide fibers (SiC_(f)_), as well as whiskers (SiC_(w)_), and alumina fibers (Al_2_O_3(f)_), have been reported. Dash et al. reported an increase in the high-temperature creep resistance of Ti_3_SiC_2_ when reinforced with SiC_(w)_ [15] and short SiC_(f)_ [16], the former presumably being due to hindered grain boundary sliding. The reinforcement of Ti_2_AlC with Al_2_O_3(f)_ also resulted in improved thermomechanical properties, with an increase in the compressive fracture strength of up to 39.7% as compared to monolithic Ti_2_AlC [17]. Furthermore, the addition of SiC_(f)_ to Cr_2_AlC led to a 70–80% increase in its wear resistance and a reduction in the friction coefficient up to 20% [18]. However, detrimental matrix/fiber interfacial reactions in the Cr_2_AlC/C_(f)_ [19], Cr_2_AlC/SiC_(f)_ [19], Ti_2_AlC/SiC_(f)_ [12,20] and Ti_3_AlC_2_/SiC_(f)_ [21] systems were documented after high-temperature operation, compromising and limiting the composite’s integrity in the long term. Therefore, a careful selection of reinforcement phase and a possible tailoring of reaction-preventing coatings [22] have to be considered. Although interfacial reaction layers were reported for Ti_2_AlC/SiC_(f)_ [12,20] and Ti_3_AlC_2_/SiC_(f)_ composites [21]—the latter leading to the formation of Ti_3_(Al_1−x_, Si_x_)C_2_ solid solutions—no apparent reaction was observed between Ti_2_AlC and Al_2_O_3(f)_ [23]. Nevertheless, the study of Spencer et al. [23] focused exclusively on reactivity between matrix and reinforcement component, lacking an investigation into the mechanical properties. In addition, large agglomerates and sintered fibers were observed. The presence of fiber agglomerates is a common issue that is observed in most studies on MAX-phase composites [19,23] employing a dry-mixing route, especially for Al_2_O_3_ fiber-reinforced systems. These can constitute areas of higher pore concentration, as they hinder sintering and, thus, curtail the composites’ performance. To date, two studies into Al_2_O_3(f)_-reinforced Ti_2_AlC are reported [17,23], among which only one focused on mechanical characterization [17]. However, the latter did not provide detailed results concerning the effect of fiber additions, and only its abstract mentioned an improvement in compressive strength.

Furthermore, the densification of MAX phases—and, more specifically, Ti_2_AlC—is known to be challenging through conventional pressureless sintering at a high temperature of 1300 °C [24,25], and even up to 1400 °C [24,26]. Sintering additives can only contribute to a limited extent to increase relative density [26] and higher sintering temperatures are undesired as they promote the decomposition of the MAX phase [27]. Methods to densify complex shapes of non-sinterable materials are not plentiful and conventional hot isostatic pressing [28] can be costly and difficult to introduce for highly porous microstructures. A concept about the low-cost densification of complex assemblies in a particulate bed of refractory materials has emerged and seems promising [29,30,31,32,33], though it is, so far, totally uncharted for MAX phases.

Therefore, more research is needed, in particular in the manufacturing of complex shapes made of MAX-phase materials. The investigation of near-net-shaping assemblies containing fibers, their optimal dispersion in the matrix, and the components’ full densification are amongst the numerous objectives sought after in the field of MAX-phase processing.

Herewith, we report, for the first time, on the manufacturing of highly homogeneous, monolithic and composite Ti_2_AlC components, reinforced with short alumina fibers by PIM, and their near-complete densification via field-sintering technology/spark-plasma sintering (FAST/SPS) in a graphite powder bed. In addition to an investigation of fiber dispersion and orientation, a detailed debinding/sintering study is presented, and macroindentation tests are performed on the final parts to evaluate their hardness properties and to compare them to existing values for non-complex shapes.

## 2. Materials and Methods

### 2.1. Material Synthesis and Processing

Ti_2_AlC powder, synthesized via the method of molten salt-shielded synthesis, described elsewhere [24], was mixed with a binder system that was optimized in a previous study [34], to produce feedstocks with 50 vol.% solid content. The binder was composed of 60 vol.% paraffin wax (Paraffin 65, Sigma-Aldrich CHEMIE GmbH, Taufkirchen, Germany), 35 vol.% of a polyethylene copolymer (Hostalen GA 7260 G, Basell Polyolefine GmbH, Frankfurt am Main, Germany), and 5 vol.% stearic acid (Merck KGaA, Darmstadt, Germany). Paraffin was used as filler to ensure the optimal flowing of the feedstock during the injection-molding procedure. Hostalen served as a backbone for injected parts and helped maintain their mechanical stability until the onset of sintering, while stearic acid was employed to decrease the feedstock’s viscosity and allow for the bridging between binder and powder [14]. The components were mixed at 170 °C for 2 h in a laboratory kneader (HKD-T 06 D, IKA-Werke GmbH & Co. KG, Staufen, Germany), equipped with duplex blades. Afterwards, the feedstock was cooled down and granulated in a cutting mill (B 06.08, Wanner Technik GmbH, Wertheim, Germany). Feedstocks containing short desized Al_2_O_3_ fibers were also produced. Fiber sizing was removed by short oxidative treatment at 700 °C for 30 min. The same mixing procedure as that described above was followed with the addition of either 1 mm hand-chopped alumina fibers (>99 wt.% Al_2_O_3_, Nextel™ 610, 3M, Maplewood, MN, USA) or 3.2 mm chopped aluminosilicate fibers (85 wt.% Al_2_O_3_ + 15 wt.% SiO_2_, Nextel™ 720, 3M, Maplewood, MN, USA). In this work, these two types of fibers are referred to as N610 and N720. Different components were molded in an injection molding machine (BOY XS, Dr. BOY GmbH, Neustadt, Germany) by using in-house produced two-part inserts, which could be adapted in the standard BOY XS mold. All components were injection molded at a pressure of 800 bar with a mold temperature of 90 °C and a screw-temperature profile varying between 175 °C near to the feed hopper and 185 °C at the nozzle. The barrel temperature was selected based on a previous work [12], as the viscosity of Ti_2_AlC-based feedstocks was found to be the lowest in this temperature range. After the injection step, the components were kept for 25 s in the mold to solidify before being ejected.

Partial binder removal (essentially paraffin wax and stearic acid were reported to dissolve [34]) ensued through solvent extraction in n-hexane at 60 °C for 48 h. Afterwards, binder burnout was carried out at 2 °C/min up to 500 °C for 2 h in a high-temperature furnace (type 121212 WM, Thermal Technology GmbH, Bayreuth, Germany), followed by a pressureless sintering (PS) step at 5 °C/min up to 1250 °C for 5 h under argon. In addition, some parts were further densified by PS in a second cycle at 5 °C/min up to 1400 °C for 3 h, so as to avoid the deformation of molybdenum plates used during the first thermal debinding/pre-sintering stage.

An additional densification step after the first PS step was also carried out by employing the approach of electroconsolidation of complex shapes [29,30,31,32,33], by using a graphite powder bed and field-assisted sintering technology/spark-plasma sintering (FAST/SPS). The parts pre-densified by PS at 1250 °C were placed in a graphite die and immersed in coarse graphite powder (−20 + 80 mesh, 99.9%, Alfa Aesar, Tewksbury, MA, USA). Graphite foil was used between die and powder, as well as between powder and Ti_2_AlC pre-sintered parts, at the top and bottom plane surfaces. In this way, mechanical load transfer and electrical and thermal contact were ensured, minimizing reactions and coarse particle imprints at the Ti_2_AlC/graphite powder interface. Powder-bed sintering (PBS) was carried out in a FAST/SPS furnace (FCT-HPD5, FCT Systeme GmbH, Rauenstein, Germany) at 100 °C/min up to 1200 °C and 50 MPa uniaxial pressure in vacuum (~4 mbar), with a dwell time of 10–15 min, depending on the size of the die (Ø 20–30 mm).

### 2.2. Material Characterization

Thermogravimetric analysis (TGA) was operated in a high-temperature DTA furnace (STA 449 F1 Jupiter, Netzsch, Selb, Germany), coupled with a mass spectrometer (QMS 403 Aeolos, Netzsch, Selb, Germany), to determine the temperature required for thermal debinding. Granulated feedstock material was heated in flowing argon up to 600 °C at a rate of 5 °C/min, using flows of 50 mL/min and 20 mL/min, respectively, to flush the furnace and protect the scale.

Vickers hardness measurements were carried out on PBS parts using a hardness tester (Duramin A-300, Struers ApS, Ballerup, Denmark) and loads of 1, 5, 10, 20 and 30 kilogram force (kgf).

Fiber dispersion and orientation were characterized using optical microscopy (OM, Axio Vert. A1, Carl Zeiss MicroImaging GmbH, Jena, Germany) coupled with a camera (AxioCam MRc, Carl Zeiss MicroImaging GmbH, Jena, Germany) and a software (AxioVision SE64 Rel. 4.9.1 SP1, Carl Zeiss Microscopy GmbH, Göttingen, Germany) and scanning electron microscopy (SEM, tabletop microscope TM3000, Hitachi High-Technologies Corporation, Tokyo, Japan). Using the MosaiX function in the AxioVision software (SE64 Rel. 4.9.1 SP1, Carl Zeiss Microscopy GmbH, Göttingen, Germany), the overview images through overlapping tiles were captured.

The relative density of the PBS parts was determined from the bulk density values measured by the Archimedes method, quantitative phase composition analysis and the rule of mixture.

Image analysis was performed on SEM and OM images of low to intermediate magnification with the image processing package Fiji for ImageJ (National Institutes of Health, Bethesda, MD, USA) to investigate the porosity of PS samples. The values reported in this work were averaged over at least 15 images to come closer to a realistic estimation of pore concentration. Fiber orientation in the (x,y) plane was determined via the software package, OrientationJ (written by Daniel Sage, Biomedical Image Group, EPFL, Lausanne, Switzerland). OM images were binarized before running the analysis. In addition, vector field and fiber orientation distribution (FOD) were determined for particular areas in injection-molded components. Furthermore, the circularity of single-fiber cross-sections was determined to deduce the orientation in the (x,z) plane. SEM images were converted to 8-bit, binarized, and major artefacts, such as alumina grains or filtered cavities. However, the presence of small clusters and porosity made the discrimination of single fibers and the elimination of artefacts upon image binarization arduous processes. Single fibers were selected, of which shape descriptors (roundness, circularity) were determined using the standard features of ImageJ.

Indents, the matrix/fiber interface and the microstructure were further characterized by SEM (Zeiss Ultra 55 and Zeiss Gemini 450, Carl Zeiss AG, Oberkochen, Germany) equipped with energy-dispersive X-ray spectroscopes (X-Max 80 mm^2^ and Ultim Max 170 mm^2^, Oxford Instruments, Abingdon, UK).

The grain size distribution of monolithic PBS specimens was determined using electron backscatter diffraction (EBSD-Camera, Nordlys II, EBSD-Software, Aztec, Oxford Instruments, Abingdon, UK) coupled to SEM (Zeiss Merlin, Carl Zeiss AG, Oberkochen, Germany). Corner and edge grains were excluded from the analysis. The grain size and aspect ratio were averaged over 1017, 377 and 108 grains of Ti_2_AlC, Ti_3_AlC_2_ and Al_2_O_3_, respectively.

## 3. Results and Discussion

### 3.1. Injection Molding and Debinding

The Ti_2_AlC powder used in this work had an irregular agglomerate shape [24]. After passing through a 500-mesh sieve, the following particle size values were obtained: D_10_ = 7.5 µm, D_50_ = 13.6 µm, and D_90_ = 22.9 µm. The distribution slope parameter S_w_ [14]—indicating the particle size distribution—was calculated according to Equation (1):

(1) $$S_{w}=\frac{2.56}{log_{10}\left(\frac{D_{90}}{D_{10}}\right)}$$

This yielded a value of ~5.3, which corresponds to a narrow particle-size distribution and allows for easier injection molding in the case of ceramic powders. However, with decreasing particle size, and due to irregular/angular particle shape, interparticle friction is more significant and feedstock viscosity increases. Accordingly, a binder content of 50 vol.% was necessary to allow for a homogeneous mixture and proper viscous flow. In addition, short desized Al_2_O_3_ fibers were used to produce composite feedstocks. According to the 3M™ Nextel™ technical reference guide, the single filament diameter was 11–14 µm for 610 and 720 fiber types. Thereby, their aspect ratio was found to vary from 80 to 270. They are composed of nanosized crystallites and were selected for their high potential compatibility with Ti_2_AlC and interesting thermo-mechanical properties, such as comparable thermal expansion (8.0 × 10^−6^ K^−1^ for N610 and 6.0 × 10^−6^ K^−1^ for N720 in the 100–1100 °C range), high melting point and operating temperature, and high filament tensile properties.

**Figure 1:**
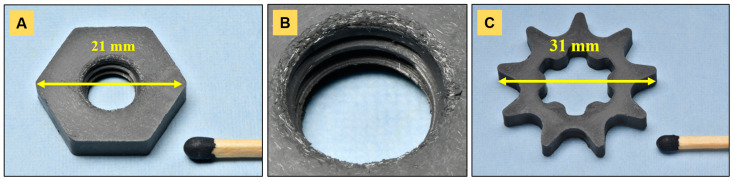
Powder-injection molded components, here containing fibers: (**A**) hexagon nut, (**B**) magnification on the threaded hole, (**C**) 9-tooth sprocket.

Figure 1 depicts the powder-injection molded parts. Standard M8 hexagonal nuts (Figure 1A) and nine-tooth sprockets (Figure 1C) with diameters of 31 mm were produced. Both components were 4 mm thick. The two-part mold inserts allowed for high shape variability in component manufacturing. In fact, nuts could be produced with and without (not shown here) a threaded hole (Figure 1B) and its size was adjustable by changing the bottom element.

Likewise, sprockets with internal geared geometry or with simple holes (not shown here) were manufactured, depending on the application tool requirements. The components were produced with minor defects—for instance, near the threaded hole (Figure 1B)—but the finish quality was high, as already reported by Gonzalez et al. [12]. In general, single cavities and cracks at the end junction between the two mass flows in the mold cavity were sometimes observed for sprockets injected from monolithic feedstocks. Fiber-containing feedstocks allowed for easier molding and less defects, probably due to better flow properties. Rapid volumetric shrinkage due to thermal contraction upon cooling caused minimal flaking at the edges during specimen removal. Injection molding was carried out in semi-automatic mode so as to place the ejected inserts back within the standard cavity after each run. However, parts could be injected at a high pace in cycles of less than 2 min.

The chemical debinding of PIM parts was a preliminary step that was essential to promoting egress of waxy binder components (paraffin wax, stearic acid) without removing the polymer backbone [14] and creating porosity to facilitate binder burnout during thermal debinding. All PIM parts lost ~11–14% of their initial mass, on account of binder extraction and slight flaking.

**Figure 2:**
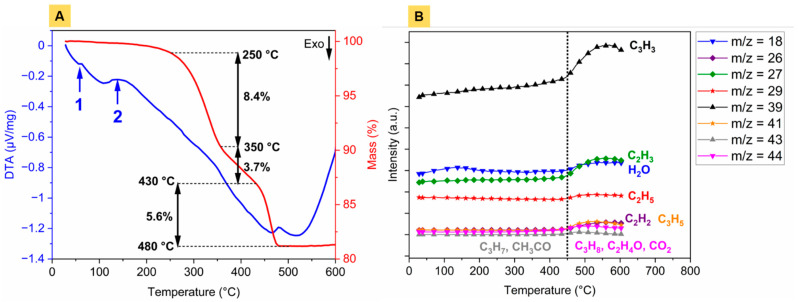
Binder burnout, analysed by (**A**) DTA/TG coupled to (**B**) mass spectrometry with corresponding QMID ion current curves. Number 1 on the DTA curve (**A**) corresponds to the melting point of paraffin wax (65 °C) and stearic acid (67 °C); number 2 represents water evaporation. Mass to charge ratio is given by m/z on (**B**).

Figure 2 highlights the features of thermal binder burnout. The DTA signal and the thermogravimetric curve (Figure 2A) demonstrated a slight mass loss (~1 wt.%) before 250 °C on account of the gradual evaporation of low-molecular-weight oligomers—most probably from residual paraffin wax and stearic acid—and adsorbed water molecules.

This was confirmed by the quasi-multiple ion detection (QMID) current curves (Figure 2B), showing a bump around 130 °C for the mass-to-charge ratio m/z = 18 (H_2_O) and a gradual increase of m/z = 39. The onset of the first decomposition segment was around 250 °C (Figure 2A). Until 350 °C, this corresponded to the major mass loss (8.4 wt.%). An intermediate decomposition stage was observed with a lower mass loss rate before reaching the third segment above 430 °C, where mass spectrometry (Figure 2B) retrieved an increase in ion current of multiple hydrocarbon compounds. The debinding temperature was set to 500 °C, at which no further sample mass variation was observed. Above this temperature, the persistence of C_x_H_y_ species (Figure 2B) in the gaseous phase would explain the retrieval of a spectrometric signal. The interest of using polyethylene as a binder constituent resides in the lowest residue weight fraction left after thermal burnout [14].

### 3.2. Pressureless Sintering

**Figure 3:**
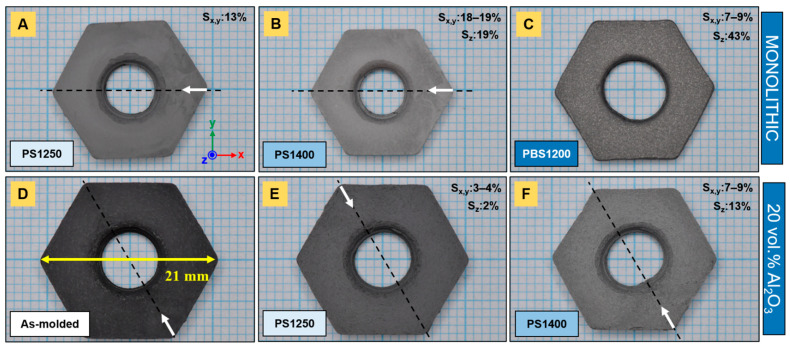
Sintered Ti_2_AlC nuts: (**A**) PS1250, (**B**) PS1400 and (**C**) PBS1200 of monolithic samples and (**D**) as-molded reference, (**E**) PS1250 and (**F**) PS1400 of N720 aluminosilicate fiber-reinforced (20 vol.%) composites. White arrows and dashed lines indicate the location of the gate and the axis parallel to the injection channel, respectively. Sintering shrinkage is given by the S values.

**Figure 4:**
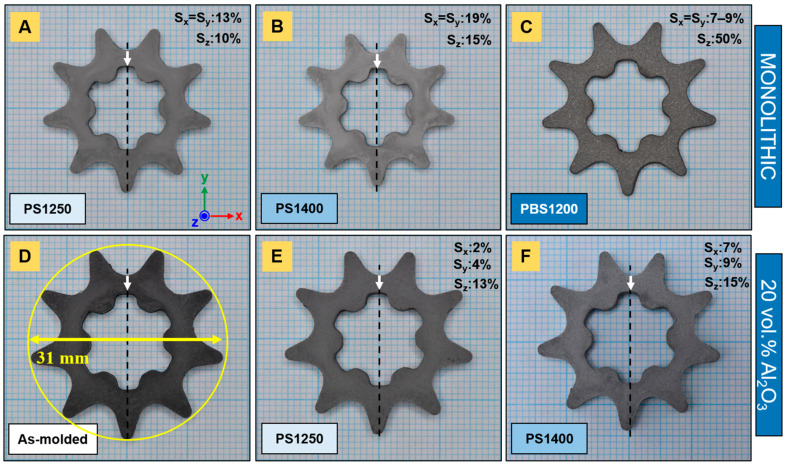
Sintered sprockets: (**A**) PS1250, (**B**) PS1400 and (**C**) PBS1200 of monolithic samples and (**D**) as-molded reference, (**E**) PS1250 and (**F**) PS1400 of N720 aluminosilicate fiber-reinforced (20 vol.%) composites. White arrows and dashed lines indicate the location of the gate and the axis parallel to the injection channel, respectively. Sintering shrinkage in each direction is given by the S values.

**Figure 5:**
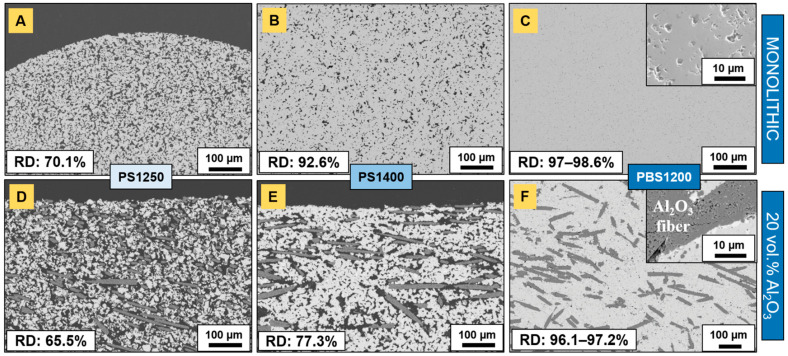
Backscattered electrons (BSE) images showing the microstructure of sintered Ti_2_AlC: (**A**) PS1250, (**B**) PS1400 and (**C**) PBS1200 of monolithic samples and (**D**) PS1250, (**E**) PS1400 and (**F**) PBS1200 of N720 aluminosilicate fiber-reinforced (20 vol.%) composites. RD designates the relative density. The insert in (C) is a secondary electrons (SE) image.

Subsequently, monolithic and composite Ti_2_AlC parts were densified by PS at different temperatures. At 1250 °C, monolithic hexagon nuts (Figure 3A), as well as sprockets (Figure 4A), experienced up to 13% isotropic shrinkage. The components achieved a relative density of 70.1% (Figure 5A) and revealed a large fraction of porosity with a high degree of interconnectivity. Recently, Tabares et al. [25] demonstrated that pressureless sintering in vacuum at 1300 °C for 4 h of injection-molded Ti_3_SiC_2_ led to porosity ranging from 47 to 53%. Up to 1300 °C in pressureless conditions, Badie et al. [24] showed that the driving force for the sintering of Ti_2_AlC is low and its densification is known to be challenging. In contrast, aluminosilicate fiber-reinforced (20 vol.% N720) composites shrank anisotropically, markedly less—around 2–4%—than their monolithic homologs. Both nuts (Figure 3E) and sprockets (Figure 4E) demonstrated minimal dimensional change compared to the as-molded components (Figure 3D and Figure 4D). The thicknesses of sprockets decreased more significantly, by 13%. The microstructural characterization of composite parts highlighted a porosity areal fraction of 34.5% (± 2.8%) (Figure 5D).

It is known that the inherent sintering ability of the MAX phases is poor without sintering additives [24,26], and temperatures as high as 1400 °C are a requirement to reach a relative density of at least 90% in PS conditions. For Ti_2_AlC, Hashimoto et al. [26] reached a theoretical density of 94.2% under pressureless conditions after 2 h at 1400 °C in argon without sintering additives; additions of Al_2_O_3_ increased the relative density to 96.0%. Lu et al. [27] proceeded to investigate the pressureless sintering of Ti_3_AlC_2_ and reported relative densities of 92.7% and 96.2% when performed at 1500 °C (10 min) and 1450 °C (150 min), respectively. Additionally, they mentioned the issue of phase decomposition into binary carbides, resulting from higher sintering temperatures or longer dwelling times.

In this work, some components were further sintered at 1400 °C by PS. The monolithic samples significantly shrank in an isotropic manner, up to 19% (Figure 3B and Figure 4B), and reached a relative density of 92.6% (Figure 5B). The microstructure highlights non-interconnected porosity and the transition to a final stage of sintering. The relatively high sintering shrinkage at 1400 °C (almost 20%) is correlated with the relatively low feedstock solids loading (50 vol.%) [14]. However, Al_2_O_3_ fiber-reinforced Ti_2_AlC composites did not achieve similar densities and shrank even less than the monolithic samples that were sintered at lower temperatures. The dimensional change in the nuts (Figure 3F) and sprockets (Figure 4F) was anisotropic. The areal fraction of pores yielded 22.7% (±1.8%) after the second PS step at 1400 °C (Figure 5E) and were mainly located in the vicinity of fibers.

Additionally, a deflection along the z-axis was observed for all parts, more pronounced for sprockets, probably due to their less compact geometry and, thus, lower bending stiffness. In particular, fiber-reinforced components became strongly undulated when sintered at 1400 °C. Similar warpage was observed for composites containing a lower volume fraction of fibers (10 vol.%), while this effect was barely visible for monolithic samples. It might find its origin in the stresses arising during injection molding, due to a more complex solidification pattern in more tortuous shapes. In fact, uneven cooling is an essential cause of warpage [35]. The longer the path that the mass followed in the sprocket mold, the larger the stress gradients and the greater the dimensional distortion upon sintering. In addition, the low solids loading (Ti_2_AlC powder, Al_2_O_3_ fibers) contributed to greater sintering shrinkage, and the presence of aligned fibers promoted the asymmetrical change in shape.

As compared to monolithic samples, the presence of a dispersed inert phase (short Al_2_O_3_ fibers) hindered sintering. In fact, the difference in the shrinkage rate of a shrinking cladding (matrix) around dispersoids leads to the buildup of an effective hydrostatic stress [36,37,38]. The latter is directly correlated with the volume fraction of the dispersed phase, when the dimensions of the reinforcing elements are significantly larger than the matrix grain size [36]. This is expressed as follows:

(2) $$\sigma_{m}=-\sigma_{i}\frac{f}{\left(1-f\right)}$$

where f and $\sigma_{i}$ are the volume fraction of the hydrostatic stress in the rigid second phase, respectively, and $\sigma_{m}$ represents the backstress generated in the matrix. As the mean hydrostatic stress or backstress $\sigma_{m}$ counteracts the sintering stress, the linear densification rate is reduced [37]. Sintering stops when the ratio of peak matrix hydrostatic stress normalized by the sintering stress approaches unity, which is the case for a volume fraction of spherical inclusions above 10% [38]. For fibers with a larger aspect ratio, shrinkage anisotropy is more pronounced [39]. In fact, due to differential shrinkage in fiber composite assemblies, the threaded hole of nuts (Figure 3E) and the internal geared geometry of sprockets (Figure 4E) became warped. The distortion was more pronounced at higher sintering temperatures (Figure 3F and Figure 4F) at which, in the case of the nut component, the initially circular threaded hole became elliptical. The thread compression induced by anisotropic sintering shrinkage was more important along the axis, parallel to the injection channel. The larger shrinkage along the sample thickness and the observed deflection are believed to result from fiber orientation, which is further discussed below. In brief, as most of the fibers were oriented in the (x,y) plane—i.e., their axis was parallel to (x,y)—their total surface area in contact with the matrix represented less opposition to shrinkage along z, as would be the case if their axes were parallel to the direction of concern.

In addition, higher processing temperatures involve the risk of phase transformation/inter-conversion from Ti_2_AlC to the more stable Ti_3_AlC_2_, as already reported by other studies [24,40], or thermal decomposition, when coming closer to the incongruent melting point of Ti_2_AlC [41]. 

In summary, we assume that the inherent low sinterability of Ti_2_AlC, the limitation in maximal sintering temperature, the absence of an additional driving force in the form of applied external pressure, the irregular agglomerate shape and the presence of rigid non-sintering fibers with high aspect ratio, did not allow for thorough densification. As such, the route of FAST/SPS in a bed of graphite powder was employed to achieve the near-complete densification of injection-molded monolithic and composite Ti_2_AlC assemblies.

### 3.3. Powder Bed Sintering

Pressureless sintering, even at an elevated temperature of 1400 °C, did not provide the required driving force to densifying the material. Instead, a pressure-assisted technique is typically required to achieve near-complete densification. Conventionally, hot isostatic pressing [28] is employed for metals and ceramics, but this is costly and often requires an encapsulation to avoid fluid penetration within the pores. Therefore, powder-bed sintering in graphite powder, using a FAST/SPS furnace [29,30,31,32,33], was found to be an alternative to achieving the near-full densification of complex shapes, as it granted the required driving force at a reduced sintering temperature (1200 °C) and dwell time (10–15 min). To the authors’ knowledge, this procedure, when applied to MAX phases, is found nowhere in the literature so far.

The pre-sintering PS step at 1250 °C was essential, as thermally debound parts exhibited high fragility and would have yielded under low loads. As compared to the PS1250 sample, a lower shrinkage percentage of ~7–9% was noticeable in the (x,y) plane (Figure 3C and Figure 4C) of the PBS parts. However, the samples significantly shrank in the z direction, which is parallel to the applied pressure. The thickness of the sprockets, sintered by PBS, was halved, while the nuts shrank up to 43%. For the latter, this was accompanied by a significant reduction in the thread pitch, while the thread diameter increased asymmetrically. In addition, subsequent sample conditioning (sandblasting and grinding), to remove the residual graphite foil and graphite particles, contributed to the decrease in thickness. The bed of coarse graphite powder may not have allowed for an optimal load transfer that was transversal to the pressing direction, so that the densification mainly occurred uniaxially. In fact, it might come from the small ratio of tool to sample size used in this work. Hocquet et al. [31] mentioned the advantage of using larger dies and its correlation with an improved repartition of the load around the sample. By convention, the transverse diameter of the powder bed should be at least twice that of the maximum transverse dimension of the body [29]. 

The relative densities of monolithic nuts and sprockets were found to be 97–97.5% and 98.1–98.6%, respectively (Figure 5C). The higher density of the latter can be ascribed to greater compaction during the injection-molding process, on account of a supposedly larger volume being “forced” into the mold cavity. The higher green densities of sprockets may have facilitated densification during sintering. The remaining 2–3% porosity was visible at higher magnification (inset of Figure 5C) and the largest pores were less than 15 µm in size. Additionally, alumina grains were found to be homogeneously distributed across the samples. Image analysis showed that these accounted for 2–3% of the considered surface areas. As for monolithic parts that are sintered by PBS, composite assemblies were almost fully densified following the same procedure (Figure 5F), albeit presenting lower relative densities than their monolithic counterparts. These were 96.1–96.5% and 96.9–97.2% of the theoretical for nuts and sprockets, respectively. From SEM and optical micrographs, it was evident that the fiber aspect ratio decreased upon feedstock granulation, as compared to the raw fibers. The maximal length was found to be less than 500 µm. Considering the diameter as unchanged (11–14 µm) and the cross-section as circular, this accounts for a maximal aspect ratio of 35–45. However, the broad fiber length distribution (as a result of fiber breakage) accounted for a broad aspect ratio variation, to the point that 100 µm fibers had an aspect ratio of 7–9. It is a well-known fact that the fractional packing density decreases with an increasing length-to-diameter (L/D) fiber ratio. For instance, a 48% random dense packing can be achieved with L/D = 10, while only a 9% fractional density is achieved with L/D = 60 [35]. Similarly, this decreases the more irregular the particle (or agglomerate) shape, as is the case here [24]. In addition, the presence of porosity within fibers (inset of Figure 5F) was observed. It occurred as soon as after the first PS step at 1250 °C and strictly concerned composites reinforced with N720 fibers, which contain 15 wt.% SiO_2_. Patches of a Si-rich phase were evidenced by EDS (not shown here) in the vicinity of fibers and were visible on backscattered electron images. It is likely that the mullite phase was decomposed at 1250 °C in a reducing atmosphere, as reported for lower temperatures [42], and led to the formation of submicrometric pores within N720 fibers. Tian et al. [42] further showed that the equilibrium total vapor pressure of mullite in argon increased by nearly four orders of magnitude from 1000 to 1250 °C and by almost six orders of magnitude when also in presence of carbon. In the present work, aside from the reducing effect of the argon atmosphere, the surrounding Ti_2_AlC matrix probably contributes to the decomposition of the 3Al_2_O_3_·2SiO_2_ phase at 1250 °C. A few cavities located nearby fibers were identified and might have been generated during sintering, as the decomposition of mullite resulted in the formation of gaseous species [42]. No such phenomenon was observed for composites reinforced by N610 alumina fibers, of which the fiber diameter remained unaltered even after PBS.

**Figure 6:**
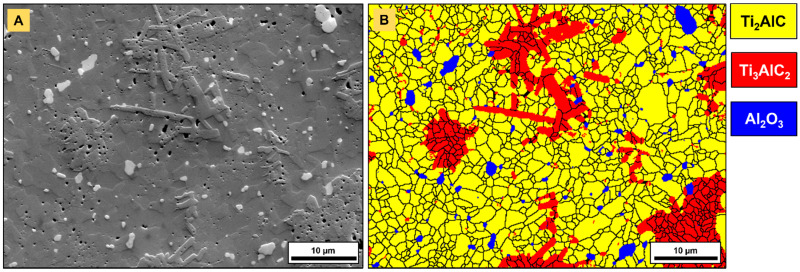
EBSD analysis of an injection molded monolithic Ti_2_AlC nut specimen after sintering procedure 1 (PS1250 + PBS1200): (**A**) SE image shows height offset and porosity in Ti_3_AlC_2_ grains and (**B**) corresponding EBSD phase map with areal fractions of 77.5% Ti_2_AlC, 18.8% Ti_3_AlC_2_ and 3.2% Al_2_O_3_.

Figure 6 highlights the outcomes of the EBSD analysis performed on a monolithic PBS specimen. The height offset of Ti_3_AlC_2_ grains, organized in clusters, as well as the presence of submicrometric pores concentrated in these specific areas, were observed (Figure 6A), as already evidenced in a previous study [24]. The presence of cavities, essentially located in and nearby to Ti_3_AlC_2_ grains, might either arise from a minor loss of Al and the interconversion of Ti_2_AlC to Ti_3_AlC_2_ [40] or from the etching of potential minor impurities (titanium aluminides) during metallographic preparation. As mentioned in a previous work [24], a major advantage of FAST/SPS is the inhibition of grain growth (Figure 6B) and the preservation of a fine-grained microstructure, as well as a limited alteration of phase composition. The alumina grains found at Ti_2_AlC grain boundaries mostly arise during the synthesis and sintering procedures [24,43] on account of a low oxygen content in raw materials or non-negligible oxygen partial pressure in furnace chambers. The average grain size yielded 1.28 µm (±0.64 µm), 0.98 µm (±0.55 µm) and 0.77 µm (±0.43 µm) for Ti_2_AlC, Ti_3_AlC_2_ and Al_2_O_3_, respectively. The phase map highlighted the broad particle size distributions for all three detected phases. In addition, the morphology of Ti_3_AlC_2_ grains was more plate-like than the Ti_2_AlC and Al_2_O_3_ grains. This was also found in the average aspect ratio, yielding 1.69 (±0.49), 1.96 (±0.79) and 1.51 (±0.38) for Ti_2_AlC, Ti_3_AlC_2_ and Al_2_O_3_, respectively.

Even though the FAST/SPS process did not enable a homogenous shape retention, high relative densities could be reached (>98%). For the first time, powder injection molded complex shapes of Ti_2_AlC MAX phase could achieve near complete densification, alongside the preservation of phase composition and grain size, via FAST/SPS in a bed of graphite powder. Dimensional changes after PBS of Al_2_O_3_ fiber-reinforced composites—independent of fiber type and volume fraction—were similar to those observed for monolithic counterparts. Unlike the differential shrinkage observed in pressureless conditions, the confinement of the samples in a carbon powder bed limited their warpage.

### 3.4. Fiber Orientation and Distribution

**Figure 7:**
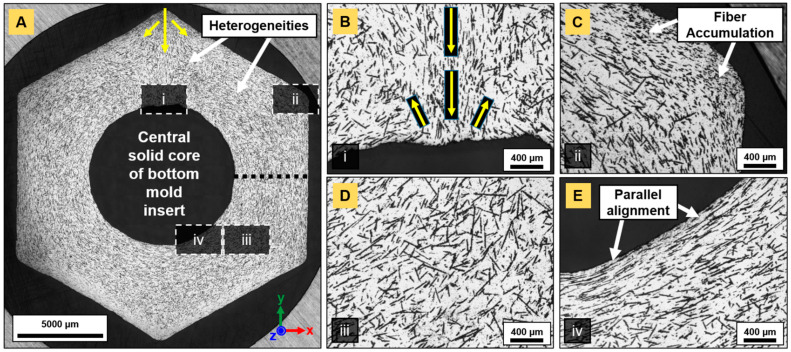
Optical micrographs showing fiber dispersion and orientation in the (x,y) plane in a PBS1200 nut reinforced with 20 vol.% N720 aluminosilicate fibers: (**A**) reconstructed image from ~140 overlapped tiles and fiber orientation in (**B**) the area opposite the mold gate, (**C**) at corners, (**D**) in bulk and (**E**) along thread. The dotted black line in (**A**) is an indication of Figure 10.

**Figure 8:**
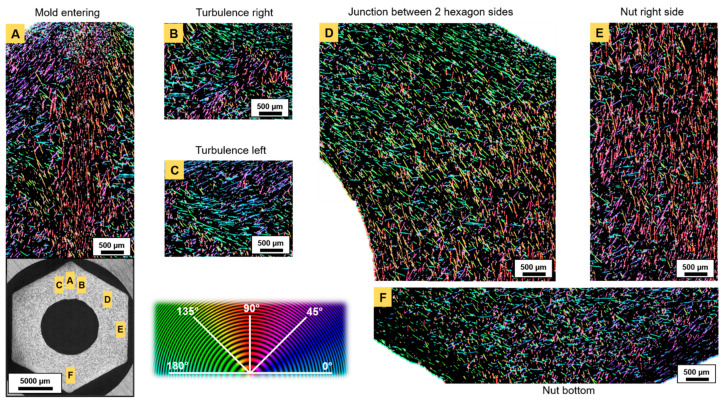
Fiber orientation pattern in a 20 vol.% fiber-reinforced Ti_2_AlC hexagonal nut: (**A**) at the mold entering, (**B**) in the right turbulence, (**C**) in the left turbulence, (**D**) at the junction between two hexagon sides, (**E**) in the right side of the nut and (**F**) in the bottom of the nut.

**Figure 9:**
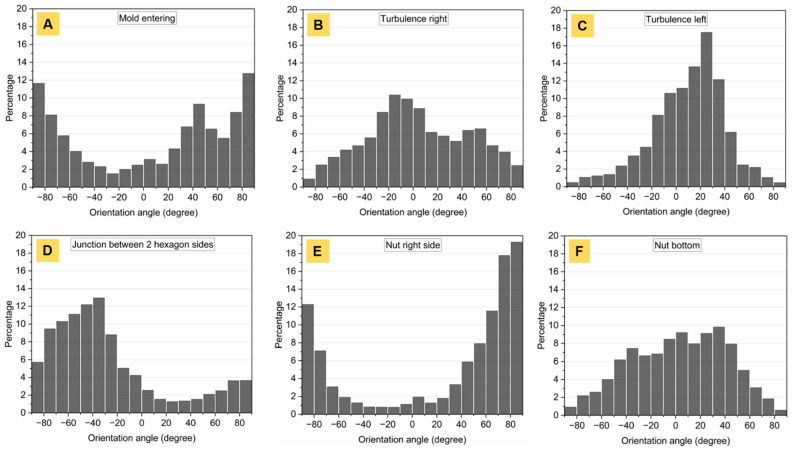
Fiber orientation distribution (FOD) in a 20 vol.% fiber-reinforced Ti_2_AlC hexagonal nut for corresponding areas in Figure 8: (**A**) at the mold entering, (**B**) in the right turbulence, (**C**) in the left turbulence, (**D**) at the junction between two hexagon sides, (**E**) in the right side of the nut and (**F**) in the bottom of the nut.

Fiber orientation and the distribution in fiber-reinforced Ti_2_AlC composites sintered by PBS were characterized in the present work (Figure 7, Figure 8 and Figure 9). The study focused on the orientation and distribution of fibers in hexagonal nuts (Figure 7 and Figure 8). These showed that, even at the highest fiber-load fraction (20 vol.%), homogeneous composites were obtained. A prevalent challenge in existing studies [15,19,23] has been the dispersion of fibers and whiskers. In this work, no agglomerates were observed, unlike those reported for Cr_2_AlC/Al_2_O_3(f)_ [19], even at lower fiber-volume fraction or Ti_2_AlC/Al_2_O_3(f)_ [23] processed via conventional dry mixing routes. The higher shear forces involved during PIM in the viscous molten material allowed for an optimal distribution of fibers in the mold cavity. It is necessary to have the reinforcement phase occupying the entire volume to avoid weak spots in the matrix. However, due to mass flow constriction, the fibers were oriented differently in each section of the injected composite assemblies. The orientation of fibers indicated the streamlines in every considered area. The constriction of the molten feedstock in the thin runner channel, upstream of the mold gate, resulted in a fiber alignment that was parallel to the runner walls. Once the molten mass penetrated the cavity (Figure 7A, yellow arrows), it essentially flowed straight towards the inner solid core of the mold insert. This is why a thin strip of material, with the fibers aligned parallel to the mold’s entering channel, was observed (area (i) in Figure 7A,B). At the mold’s entering channel (Figure 8A), the color hue indicated a 90° orientation of the fibers and two side flows with a dominant +45° and −45° (+135°) orientation. The corresponding fiber orientation distribution (FOD) (Figure 9A) confirmed the ±90° dominant direction, in which fibers were aligned, in addition to a high percentage of fibers in a +45° direction. Once the mass reached the central solid cylinder of the mold insert, two sideways streams (Figure 7B and Figure 8A–C) were observed in the vicinity of the oriented strip.

As a result of the compression between the downwards entering mass flow and the opposite upwards stream, the FOD were quasi-monomodal (Figure 9B,C). This indicates that most of the fibers were oriented perpendicular to the entering flow. In segments parallel to the injection channel (Figure 8E) and in all four of the other sides of the hexagonal nut (Figure 8D), the dominant orientations were ±90° and −45°, respectively (Figure 9E,D), indicating the good alignment of fibers with the flow. At corners (area (ii) in Figure 7A,C), fibers were essentially oriented in the z-direction, where their quasi-circular cross-section was visible. In the central ring (area (iii) in Figure 7A,D), the orientation pattern was more random, while in nearby mold walls (area (iv) in Figure 7A,E and Figure 8E), the fibers were aligned in the (x,y) plane along the edges.

In each section of the nut mold cavity, an understanding of the model of viscous flow between two parallel plates can be established [44,45] with a quasi-parabolic mass flow velocity profile. Due to higher shear stress in the near-wall regions, the flow velocity is lower. In the centerline, the flow velocity reaches a maximum. In addition, velocity gradients are higher at mold walls and are lower in the center [46]. This explains the random orientation of the fibers in the center and their parallel alignment near the wall, as stronger velocity gradients significantly act on the rotational motion of fibers. In turn, the hydrodynamic drag force created on fibers aligned alongside walls increased. Further, Marchioli et al. [46] reported fiber deposition and accumulation in near-wall regions. In the present work, such outcomes were noticed at corners of the nut-shaped mold (Figure 7C). A higher density of fibers was observed in these areas, probably retained by larger frictional forces. Their preferential orientation towards the z-axis was deduced from optical micrographs, as their cross-section could mostly be identified. The first fibers reaching the walls may have been pinned at one end and, due to the pressure exerted by the flowing material filling the cavity, may have been subjected to torque, inducing their rotation and dominant orientation along the orthogonal (x,z) plane. The viscous material in the near-wall regions (in direct contact with the colder mold) is known to cool faster due to heat dissipation, and builds up a thin solidified layer, leaving the mass in a frozen state [35]. Heterogeneities in the bottom area of the nut (Figure 8F) were observed, which resulted from mass flows in both last ±45° sections meeting and causing rotation of fibers. There was no dominant orientation as fibers were equally aligned in different directions and the FOD indicated a broad distribution over a large angle range (Figure 9F).

**Figure 10:**
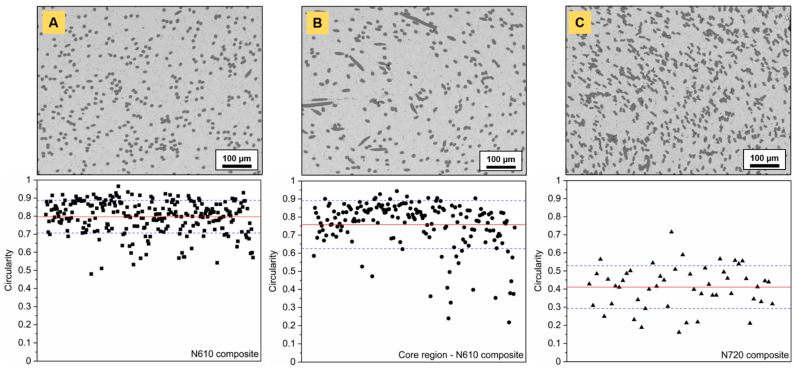
Circularity of fiber cross-sections determined for: (**A**) 10 vol.% N610 fiber-reinforced, (**B**) 10 vol.% N610 fiber-reinforced (core region) and (**C**) 20 vol.% N720 fiber-reinforced Ti_2_AlC composites.

The analysis of circularity is given in Figure 10 and was performed on N610 and N720-containing composites. The cross-sections were taken at the nut’s mid-plane, as indicated by the dotted black line in Figure 7A and highlighted a homogeneous fiber distribution in the entire mold volume. Circularity is calculated as follows:

(3) $$C=4\pi \frac{A}{P^{2}}$$

with A as the area and P as the perimeter of the fiber’s cross-section. The closer to 1, the more circular the analyzed element. N610 Al_2_O_3_ fibers (Figure 10A) presented almost no variation in fiber diameter after sintering, as the average circularity determined from 260 fiber cross-sections yielded 0.797 ± 0.091. It also confirmed that fiber axes were orthogonal to the sectional plan, i.e., that fibers were mostly oriented parallel to the flow. The same analysis performed in the composite’s core (Figure 10B) highlighted minima in circularity down to ~0.2, indicating fibers with more elongated shape, as visible on the SEM image. 

The average circularity decreased to 0.758 ± 0.133. The core might have been subjected to turbulent flow, leading to the formation of vortices. These vortex-like patterns were observed for all fiber-composite types and at various fiber volume fractions. Essentially, they may have acted on the rotational motion of fibers, the latter axes of which were found to be parallel to the cutaway plane. The analysis of circularity for N720 fiber-reinforced composites (Figure 10C) was challenging due to the higher fiber content. Nevertheless, the decrease in circularity (0.411 ± 0.118) in areas around the core region was not due to an alignment of fibers perpendicular to the flow, but rather caused by their prolate cross-section, discussed earlier.

However, these outcomes provided clues for the prediction of shrinkage anisotropy. The consideration of the overall FOD, which was averaged over each individual section of the nut composite assemblies, indicated that the mean dominant direction was favorable to a greater shrinkage along the injection axis. In fact, only the two sides parallel to the injection axis and the strip at the mold entering genuinely appeared as a hindrance to sintering, while all four of the other segments were less counteracting densification. An analogous reasoning could be established for sprocket assemblies.

### 3.5. Mechanical Characterization

**Figure 11:**
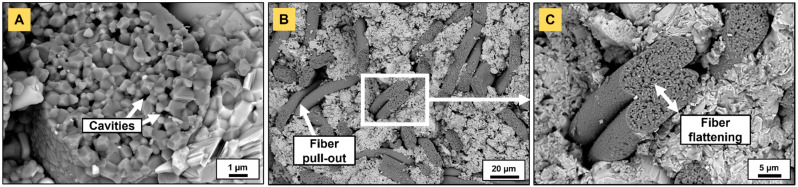
Fracture surface of a Ti_2_AlC nut reinforced with 20 vol.% N720 fibers: (**A**) submicrometric pores arising from mullite decomposition, (**B**) partial fiber pullout observed at (**C**) higher magnification with fiber flattening.

Outcomes of the fractographic analysis of a Ti_2_AlC/N720 composite are depicted in Figure 11. Figure 11A highlights the presence of submicrometric cavities that are generated by the decomposition of the mullite phase. Limited fiber pullout—the length of which was approximately 15–20 µm for the considered area—was observed (Figure 11B,C), as for Cr_2_AlC/Al_2_O_3(f)_ [19], and may have contributed, to a minimal extent, to the overall strain energy-absorbing behavior of the composite. The absence of matrix/fiber interfacial coating usually results in a fairly strong interface, and the fracture surfaces of Al_2_O_3_ fiber-reinforced Ti_2_AlC generally demonstrated neat cuts throughout the matrix and fibers. Here, the near-fiber porosity may have facilitated interfacial crack propagation and fiber sliding, as it directly affects the work for debonding and the work to pullout. Fiber flattening (Figure 11C) may have resulted from the decrease in strength induced by the decrease in the amount of mullite and the generation of porosity. As mentioned earlier, this was observed after the first PS step at 1250 °C, at which temperature N720 fibers must have plastically deformed. Typically, they exhibited an elliptical cross-section with a major axis of 20 µm and a minor axis of 8 µm, deviating from the initial circular cross-section with Ø 12–14 µm.

**Figure 12:**
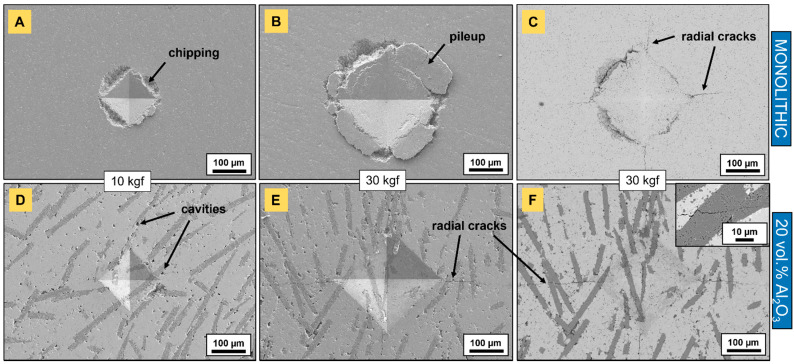
Vickers indentations on powder-bed-sintered monolithic and aluminosilicate fiber-reinforced (20 vol.%) Ti_2_AlC composites. (**A**–**E**) are SE images, and (**C**,**F**) are BSE images.

Vickers indentation (Figure 12) was carried out at different loads to evaluate the response of monolithic and fiber-reinforced Ti_2_AlC composites. At loads of up to a 10-kilogram force (~98 N), no radial cracks were visible for monolithic samples (Figure 12A). Instead, the chipping of the deformed material was observed—and started to appear at loads of 5 kgf (~49 N), probably as a result of subsurface crack propagation around the indent and subsequent collective grain pullout. This flaking was more pronounced with increasing applied load, as also reported by Amini et al. [47] for Ti_2_SC, due to it having a larger pseudo-plastic zone. These outcomes are in agreement with one of the hallmarks of MAX phases—their damage tolerance [2,47,48] as a result of basal slip activation. At higher loads (30 kgf), monolithic Ti_2_AlC accommodated the stresses in the form of massive material pileup (Figure 12B). This mechanism is well known for MAX phases and takes place as the material absorbs the strain energy through the formation of kink-bands, delamination, and buckling [48,49]. Radial cracks propagating from the four corner points of the indents were observed for applied loads of 20 kgf (~196 N) and 30 kgf (~294 N) (Figure 12C). They belong to the Palmqvist type of crack as their crack-to-indent ratio size $\left(\frac{l}{a}\right)$ was below 2.5 [50,51]. These have not usually been observed for coarse-grained monolithic MAX phases, such as Ti_3_SiC_2_ [48], Ti_2_SC [47] or Cr_2_AlC [18]. However, fine-grained Ti_2_SC presented radial cracks that started to appear from loads of 50 N, indicating its greater brittleness compared to its coarse-grained Ti_2_SC counterparts [47] and Ti_2_AlC with the equivalent grain size from the present work.

From the apparent crack length, a rough estimation of the apparent toughness *K_IC_* could be made using the following formula [50]:

(4) $$\frac{K_{IC}\Phi }{Ha^{\frac{1}{2}}}{\left(\frac{H}{E\Phi }\right)}^{\frac{2}{5}}=0.048{\left(\frac{l}{a}\right)}^{-\frac{1}{2}}$$

with *Φ* as a constraint factor (~3), *H* as the hardness, *E* (=277 GPa [52]) representing the Young’s modulus, *a* as the half-diagonal of the indent and *l* representing the Palmqvist crack length. The apparent fracture toughness yielded 10.04 ± 0.47 MPa·m^1/2^ for pure Ti_2_AlC. This value is within the range of *K_IC_*, reported for various MAX phases [2], though is higher than that measured by a single-edge notched beam for coarse-grained Ti_2_AlC (6.5 MPa·m^1/2^) [53] and fine-grained Ti_2_AlC_0.7_ (6.5 MPa·m^1/2^) [54]. It has to be emphasized that the toughness determined in this work accounts for Ti_2_AlC with ancillary phases of Ti_3_AlC_2_ and Al_2_O_3_ (Figure 6) and solely provides an estimation of the critical stress intensity factor. As the formula contained approximations, such as the *Φ* and *E* modulus, retrieved from other studies, measured crack length diverging from the real crack length and the consideration of a Palmqvist crack regime, the accuracy of the calculated value is less reliable than conventional fracture toughness measurement methods.

While monolithic samples clearly exhibited energy-absorbing mechanisms such as material chipping (Figure 12A) and pileup (Figure 12B), these were inhibited in the presence of fibers (Figure 12D–F). While chipping was sometimes observed for indentations present in the bare Ti_2_AlC matrix (not shown here), material delamination, pileup and flaking off were not observed when the indenter tip hit fibers. Instead, the indentations were perfectly pyramidal. The near-fiber cavities that were mentioned earlier were evidenced on secondary electron SEM images (Figure 12D,E). Radial cracks emanating from indentation corners were observed at lower loads (5 kgf, ~49 N) than for their monolithic counterparts at 30 kgf (~294 N), which can be seen in Figure 12E,F. The presence of cracks at lower loads justified the increase in brittleness of Ti_2_AlC composites as compared to their monolithic homolog, due to the incorporation of a large volume fraction of the brittle dispersoid phase. Likewise, these radial cracks were reported for Cr_2_AlC/SiC_(f)_ systems [18] at loads of 5 and 20 kgf. The apparent toughness was estimated the same as for monolithic samples and yielded 7.90 ± 0.66 MPa·m^1/2^. The decrease in toughness can be explained by two joint facts: the addition of stiff ceramic fibers curbing the energy-absorbing abilities of Ti_2_AlC and the strong bonding between the fiber and matrix, not allowing cracks to be deflected at the interface. In fact, in general, through-fiber crack propagation was observed (inset in Figure 12F) with limited deflection. Even though the addition of alumina fibers without coating or tailoring of the interface/interphase did not promote an increase in toughness, it is expected that the overall strength, for example, in compression, can be improved [17], and that creep rates could potentially be reduced.

The evolution of Vickers hardness versus applied indentation load is given in Table 1. In general, little influence of fiber addition on the hardness was observed, as the values remained within the range of the standard deviation. These fall into the 2–8 GPa range, known for polycrystalline MAX phases [2]. They were mostly comprised of between 5.5 GPa and 6.7 GPa and were higher than those reported for coarse-grained Ti_2_AlC [2,43,53,55]. A closer hardness value (5.8 GPa) was reported for fine-grained (~6 µm) non-stoichiometric Ti_2_AlC_0.7_ [54]. At low loads, the surface area of indents was small, and the average diagonal length yielded 54 µm and 123 µm for 1 kgf and 5 kgf, respectively. Therefore, hardness values in the case of composite samples with low to intermediate fiber volume fraction cannot be as accurate as for monolithic Ti_2_AlC. These are rather an average representation of hardness, with selective consideration for either the bare Ti_2_AlC matrix or of single alumina fibers. From 10 kgf, the surface area of indents was large enough to provide a realistic estimation of composite hardness (average diagonal length was 172 µm) and the maximum average diagonal length of indents produced at 30 kgf yielded 289 µm. A slight increase in hardness was measured at the lowest fiber volume fraction (5 vol. %), while it decreased for the highest content (20 vol. %). The latter can be explained by the lowest densities which were reached for composites with the significant addition of fibers. Furthermore, with a finer grain size distribution, the decrease in Vickers hardness with an applied load is less marked [2], as compared to coarse-grained microstructures. This is why, in most cases, hardness curves tended towards an asymptote, as demonstrated for Ti_2_SC [47].

## 4. Summary and Conclusions

This paper demonstrates the injection molding of monolithic and alumina fiber-reinforced Ti_2_AlC, followed by densification steps, as well as the characterization of mechanical properties. Powder-injection molding was found to be a powerful technique to rapidly produce near-net shape hexagonal nuts and sprockets with limited defects. The variability in mold design allowed for a higher degree of freedom in the manufacturing of complex shapes. A solids loading of 50 vol.% was employed to maintain the viscosity within the range of moldable feedstocks. After binder removal, the pre-sintering step at 1250 °C without external pressure resulted in highly porous samples with relative densities ranging from 65 to 70%. Pressureless sintering at 1400 °C notably increased the relative density of monolithic parts to ~93%, but fiber composites experienced few changes, especially when high fiber content was used. Thereof, the relative density varied between ~65% to ~77% in the 1250–1400 °C range. While monolithic components shrank isotropically at both investigated temperatures, the differential sintering shrinkage of composite parts led to significant warpage at 1400 °C. The electric field and pressure-assisted consolidation in a bed of graphite powder provided a sufficient driving force at 1200 °C and within 10 min to densifying both types of samples to almost 99% the theoretical value, while preserving the phase composition and fine-grained microstructure. The distribution of fibers was excellent, even in composites containing a large volume fraction of reinforcement (20 vol. %). These were essentially aligned in the direction of the flow. Specific areas, such as at the mold entering, at the corners and in the specimen’s core, were also subject to heterogeneities. The pressureless sintering of composites at 1250 °C resulted in a 4% and 3% differential shrinkage along the axes parallel and perpendicular to the injection channel, respectively. At 1400 °C, the overall fiber orientation distribution led to a 10% and 7% shrinkage along these axes, leading to visible asymmetrical dimensions. In addition, the reducing atmosphere during the sintering procedure caused a decomposition of the mullite phase contained in the Nextel™ 720 alumina fibers, and resulted in the formation of micro-cavities within alumina. In turn, the strength of fibers was reduced, and their cross-section became elliptical due to compression. Fractured surfaces did not show significant fiber pullout—the length of which was in the order of 15–20 µm—as the strong bonding between matrix and fibers facilitated through-fiber crack propagation, instead of interfacial deflection. Indentation showed that the addition of fibers did not contribute to increasing the material’s hardness, while monolithic samples demonstrated energy-absorbing mechanisms, such as material pileup, chipping, or absence of cracks up to intermediate loads. The decreased toughness of composites is assumed to be caused by: (1) the addition of stiff ceramic fibers inhibiting the energy-absorbing abilities of Ti_2_AlC and (2) the strong bonding between the fibers and the matrix not enabling crack deviation at the interface.

For the first time, monolithic and fiber-containing MAX phase assemblies with complex geometries produced by powder injection molding have been successfully sintered to near complete densification via field-assisted sintering technology/spark-plasma sintering (FAST/SPS) in a bed of graphite powder. This will pave the way in the rapid manufacturing of near-net shape MAX-phase components and the control—so far challenging—of their final density.

Even though the incorporation of alumina fibers did not increase either the hardness or toughness, it is expected that the strength in compression and creep can be improved. The design of fiber-reinforced MAX-phase composites with the tailoring of suitable coatings for alumina fibers would be an interesting track to pursue. Future optimization studies should focus on the control of the sintering procedure, as it appears to be the key step in avoiding mullite-containing fiber decomposition. Additionally, mold design considerations, such as the determination of cavity-expansion coefficients [14], could help to maintain homogeneous dimensions.

## Figures and Tables

**Table 1:** Vickers hardness of powder bed sintered monolithic and Al_2_O_3_ fiber-reinforced Ti_2_AlC composites. Vickers hardness is given for loads of 1, 5, 10, 20 and 30 kgf. Blank values for HV1, 5 and 20 are account of non-pyramidal indentations.

Samples	Hardness (GPa)					Apparent K_IC_ (MPa·m^1/2^)
	HV1	HV5	HV10	HV20	HV30	
Monolithic	6.14 (±0.31)	5.82 (±0.35)	6.16 (±0.45)	5.92 (±0.49)	5.87 (±0.54)	10.04 (±0.47)
5 vol.% N720	N/A	N/A	6.67 (±0.59)	6.17 (±0.15)	6.13 (±0.32)	N/A
10 vol.% N610	6.21 (±0.77)	6.17 (±0.34)	5.95 (±0.43)	N/A	5.79 (±0.32)	N/A
20 vol.% N720	5.51 (±0.39)	5.89 (±0.38)	5.52 (±0.34)	5.62 (±0.35)	5.39 (±0.16)	7.90 (±0.66)

## Data Availability

Data sharing not applicable.

## References

[1] Gonzalez-Julian J. (2021). Processing of MAX phases: From synthesis to applications. J. Am. Ceram. Soc..

[2] Barsoum M.W. (2013). MAX Phases: Properties of Machinable Ternary Carbides and Nitrides.

[3] Bai Y., Kong F., He X., Li N., Qi X., Zheng Y., Zhu C., Wang R., Duff A. (2017). Thermal shock behavior of Ti_2_AlC from 200 to 1400 °C. J. Am. Ceram. Soc..

[4] Tallman D.J., Anasori B., Barsoum M.W. (2013). A Critical Review of the Oxidation of Ti_2_AlC, Ti_3_AlC_2_ and Cr_2_AlC in Air. Mater. Res. Lett..

[5] Wang X.H., Zhou Y.C. (2003). High-Temperature Oxidation Behavior of Ti_2_AlC in Air. Oxid. Met..

[6] Basu S., Obando N., Gowdy A., Karaman I., Radovic M. (2011). Long-Term Oxidation of Ti_2_AlC in Air and Water Vapor at 1000–1300 °C Temperature Range. J. Electrochem. Soc..

[7] Smialek J.L., Cuy M.D., Harder B.J., Garg A., Rogers R.B. (2020). Durability of YSZ coated Ti_2_AlC in 1300 °C high velocity burner rig tests. J. Am. Ceram. Soc..

[8] Belmonte M., Koller M., Moyano J.J., Seiner H., Miranzo P., Osendi M.I., González-Julián J. (2019). Multifunctional 3D-Printed Cellular MAX-Phase Architectures. Adv. Mater. Technol..

[9] Nan B., Yin X., Zhang L., Cheng L. (2011). Three-Dimensional Printing of Ti_3_SiC_2_-Based Ceramics. J. Am. Ceram. Soc..

[10] Ma Y., Yin X., Fan X., Wang L., Greil P., Travitzky N. (2015). Near-Net-Shape Fabrication of Ti_3_SiC_2_-based Ceramics by Three-Dimensional Printing. Int. J. Appl. Ceram. Technol..

[11] Elsayed H., Chmielarz A., Potoczek M., Fey T., Colombo P. (2019). Direct ink writing of three dimensional Ti_2_AlC porous structures. Addit. Manuf..

[12] Gonzalez-Julian J., Classen L., Bram M., Vaßen R., Guillon O. (2016). Near Net Shaping of Monolithic and Composite MAX Phases by Injection Molding. J. Am. Ceram. Soc..

[13] Stumpf M., Fan X., Biggemann J., Greil P., Fey T. (2019). Topological interlocking and damage mechanisms in periodic Ti_2_AlC-Al building block composites. J. Eur. Ceram. Soc..

[14] German R.M., Bose A. (1997). Injection Molding of Metals and Ceramics.

[15] Dash A., Malzbender J., Dash K., Rasinski M., Vaßen R., Guillon O., Gonzalez-Julian J. (2020). Compressive creep of SiC whisker/Ti_3_SiC_2_ composites at high temperature in air. J. Am. Ceram. Soc..

[16] Dash A., Malzbender J., Vaßen R., Guillon O., Gonzalez-Julian J. (2020). Short SiC fiber/Ti_3_SiC_2_ MAX phase composites: Fabrication and creep evaluation. J. Am. Ceram. Soc..

[17] Naik Parrikar P., Gao H., Radovic M., Shukla A. (2015). Static and Dynamic Thermo-Mechanical Behavior of Ti_2_AlC MAX Phase and Fiber Reinforced Ti_2_AlC Composites. Conference Proceedings of the Society for Experimental Mechanics Series.

[18] Gonzalez-Julian J., Llorente J., Bram M., Belmonte M., Guillon O. (2017). Novel Cr_2_AlC MAX-phase/SiC fiber composites: Synthesis, processing and tribological response. J. Eur. Ceram. Soc..

[19] Go T., Vaßen R., Guillon O., Gonzalez-Julian J. (2021). Processing and oxidation response of Cr_2_AlC MAX-phase composites containing ceramic fibers. Open Ceram..

[20] Spencer C.B., Córdoba J.M., Obando N.H., Radovic M., Odén M., Hultman L., Barsoum M.W. (2011). The Reactivity of Ti_2_AlC and Ti_3_SiC_2_ with SiC Fibers and Powders up to Temperatures of 1550 °C. J. Am. Ceram. Soc..

[21] Guo S., Hu C., Gao H., Tanaka Y., Kagawa Y. (2015). SiC(SCS-6) fiber-reinforced Ti_3_AlC_2_ matrix composites: Interfacial characterization and mechanical behavior. J. Eur. Ceram. Soc..

[22] Guo S. (2016). Improvement of mechanical properties of SiC(SCS-6) fibre-reinforced Ti_3_AlC_2_ matrix composites with Ti barrier layer. J. Eur. Ceram. Soc..

[23] Spencer C.B., Córdoba J.M., Obando N., Sakulich A., Radovic M., Odén M., Hultman L., Barsoum M.W. (2011). Phase Evaluation in Al_2_O_3_ Fiber-Reinforced Ti_2_AlC during Sintering in the 1300–1500 °C Temperature Range. J. Am. Ceram. Soc..

[24] Badie S., Dash A., Sohn Y.J., Vaßen R., Guillon O., Gonzalez-Julian J. (2021). Synthesis, sintering, and effect of surface roughness on oxidation of submicron Ti_2_AlC ceramics. J. Am. Ceram. Soc..

[25] Tabares E., Cifuentes S.C., Jiménez-Morales A., Tsipas S.A. (2021). Injection moulding of porous MAX phase Ti_3_SiC_2_ without using space-holder. Powder Technol..

[26] Hashimoto S., Takeuchi M., Inoue K., Honda S., Awaji H., Fukuda K., Zhang S. (2008). Pressureless sintering and mechanical properties of titanium aluminum carbide. Mater. Lett..

[27] Lu X., Zhou Y. (2010). Pressureless Sintering and Properties of Ti_3_AlC_2_. Int. J. Appl. Ceram. Technol..

[28] Helle A., Easterling K., Ashby M. (1985). Hot-isostatic pressing diagrams: New developments. Acta Metall..

[29] Lange F.F., Terwilliger G.R. (1972). Method of Compacting Shaped Powdered Objects. U.S. Patent.

[30] Lichti W.P., Hofstatter A.F. (1985). Method of Object Consolidation Employing Graphite Particulate. U.S. Patent.

[31] Hocquet S., Dupont V., Cambier F., Ludewig F., Vandewalle N. (2020). Densification of complex shape ceramics parts by SPS. J. Eur. Ceram. Soc..

[32] Goldberger W.M. (1993). Method for Electroconsolidation of a Preformed Particulate Workpiece. U.S. Patent.

[33] Goldberger W., Merkle B.D. (2001). Electroconsolidation offers fast, low-cost densification. Met. Powder Rep..

[34] Barbosa A.P.C., Bram M., Stöver D., Buchkremer H.P. (2013). Realization of a Titanium Spinal Implant with a Gradient in Porosity by 2-Component-Metal Injection Moulding. Adv. Eng. Mater..

[35] German R.M. (1990). Powder Injection Molding.

[36] Weiser M.W., De Jonghe L.C. (1988). Inclusion Size and Sintering of Composite Powders. J. Am. Ceram. Soc..

[37] Rahaman M.N. (2007). Sintering of Ceramics.

[38] Hsueh C.-H. (1986). Sintering behaviour of powder compacts with multiheterogeneities. J. Mater. Sci..

[39] Stedman S., Evans J., Brook R., Hoffmann M.J. (1993). Anisotropic sintering shrinkage in injection-moulded composite ceramics. J. Eur. Ceram. Soc..

[40] Kisi E.H., Wu E., Zobec J.S., Forrester J.S., Riley D.P. (2007). Inter-Conversion of M*_n_*_+1_AX*_n_* Phases in the Ti-Al-C System. J. Am. Ceram. Soc..

[41] Pang W., Low I., O’Connor B., Peterson V., Studer A., Palmquist J. (2011). In situ diffraction study of thermal decomposition in Maxthal Ti_2_AlC. J. Alloys Compd..

[42] Tian J., Shobu K. (2004). Fracture strength of melt-infiltrated SiC-mullite composite. J. Mater. Sci..

[43] Barsoum M.W., El-Raghy T., Ali M. (2000). Processing and characterization of Ti_2_AlC, Ti_2_AlN, and Ti_2_AlC_0.5_N_0.5_. Metall. Mater. Trans. A.

[44] Tadmor Z. (1974). Molecular orientation in injection molding. J. Appl. Polym. Sci..

[45] Gogos C.G., Huang C.-F., Schmidt L.R. (1986). The process of cavity filling including the fountain flow in injection molding. Polym. Eng. Sci..

[46] Marchioli C., Fantoni M., Soldati A. (2010). Orientation, distribution, and deposition of elongated, inertial fibers in turbulent channel flow. Phys. Fluids.

[47] Amini S., Barsoum M.W., El-Raghy T. (2007). Synthesis and Mechanical Properties of Fully Dense Ti_2_SC. J. Am. Ceram. Soc..

[48] El-Raghy T., Zavaliangos A., Barsoum M.W., Kalidindi S.R. (2005). Damage Mechanisms around Hardness Indentations in Ti_3_SiC_2_. J. Am. Ceram. Soc..

[49] Zhou A.G., Barsoum M.W., Basu S., Kalidindi S., Elraghy T. (2006). Incipient and regular kink bands in fully dense and 10 vol.% porous Ti_2_AlC. Acta Mater..

[50] Niihara K. (1983). A fracture mechanics analysis of indentation-induced Palmqvist crack in ceramics. J. Mater. Sci. Lett..

[51] Liang K.M., Orange G., Fantozzi G. (1990). Evaluation by indentation of fracture toughness of ceramic materials. J. Mater. Sci..

[52] Hettinger J.D., Lofland S.E., Finkel P., Meehan T., Palma J., Harrell K., Gupta S., Ganguly A., El-Raghy T., Barsoum M.W. (2005). Electrical transport, thermal transport, and elastic properties of *M*_2_AlC (*M* = Ti, Cr, Nb, and V). Phys. Rev. B.

[53] Wang X., Zhou Y. (2002). Solid-Liquid Reaction Synthesis and Simultaneous Densification of Polycrystalline Ti_2_AlC. Z. Met..

[54] Bai Y., He X., Li Y., Zhu C., Zhang S. (2009). Rapid synthesis of bulk Ti_2_AlC by self-propagating high temperature combustion synthesis with a pseudo-hot isostatic pressing process. J. Mater. Res..

[55] Barsoum M.W., Brodkin D., El-Raghy T. (1997). Layered machinable ceramics for high temperature applications. Scr. Mater..

